# Editorial: Immune and autoimmune mechanisms in cardiovascular disease

**DOI:** 10.3389/fcvm.2022.1125959

**Published:** 2023-01-09

**Authors:** Holger Winkels, Norbert Gerdes, Florian Kahles, Partha Dutta, Chiara Giannarelli, Dennis Wolf

**Affiliations:** ^1^Department of Cardiology, Faculty of Medicine and University Hospital Cologne, University of Cologne, Cologne, Germany; ^2^Division of Cardiology, Pulmonology, and Vascular Medicine, Medical Faculty, University Hospital, Heinrich-Heine University, Düsseldorf, Germany; ^3^Medical Faculty, Cardiovascular Research Institute Düsseldorf (CARID), Heinrich-Heine University, Düsseldorf, Germany; ^4^Department of Cardiology, Angiology and Intensive Care Medicine, University Hospital Aachen, Aachen, Germany; ^5^Division of Cardiology, Department of Medicine, Pittsburgh Heart, Lung, Blood and Vascular Medicine Institute, University of Pittsburgh School of Medicine, Pittsburgh, PA, United States; ^6^Division of Cardiology, Department of Medicine, New York University (NYU) Grossman School of Medicine, New York, NY, United States; ^7^Division of Cardiology, Department of Pathology, New York University (NYU) Grossman School of Medicine, New York, NY, United States; ^8^Cardiology and Angiology, Medical Center, University Heart Center and Faculty of Medicine, University of Freiburg, Freiburg im Breisgau, Germany

**Keywords:** inflammation, immunity, autoimmunity, atherosclerosis, cardiovascular, T cells, B cells

Atherosclerosis and its sequelae, myocardial infarction, ischemic heart disease, and stroke remain the leading cause of mortality worldwide. A large body of clinical and pre-clinical evidence has suggested that cardiovascular disease (CVD) is driven by a chronic inflammatory response in arteries and the ischemic heart that leads to an accumulation of leukocytes in affected tissues. Inflammation in the cardiovascular system is accompanied by an autoimmune response involving T- and B-lymphocytes recognizing atherosclerosis-relevant autoantigens in arteries and myocardium-specific autoantigens in the heart. Although it is clinically well-established that cardiometabolic conditions, such as hypertension, diabetes, and obesity, are linked to chronic low-grade inflammation and enhance the risk for CVD, the underlying precise mechanisms remain poorly defined. While inflammation in some CVD entities is already therapeutically modifiable, for instance by canakinumab, an antibody neutralizing the pro-inflammatory cytokine IL-1β or colchicine, autoimmunity remains an unresolved clinical problem. However, preclinical models suggest that cardiovascular autoimmunity may be addressable by novel immunomodulation or tolerogenic vaccination with autoantigens to boost the protective limbs of autoimmunity and limit inflammation. Such strategies promise specific and causal therapeutic interventions that may overcome side-effects of unspecific anti-inflammatory therapies. Exact triggers, antigens, and dynamics of this immune response as well as potential targets for future clinical therapies, however, remain only partially understood. Here, we present a series of articles highlighting novel aspects of the (auto-) immune and inflammatory response in CVD.

The recruitment of leukocytes into tissues represents a hallmark of inflammation. Leukocyte migration into atherosclerotic plaques or the heart is orchestrated by a complex interplay of chemokine-, integrin-, and selectin receptors and ligand pairs. Our series starts with Mauersberger et al. summarizing current concepts of leukocyte recruitment into the atherosclerotic aorta with a specific emphasis on cell types and therapeutically addressable recruitment pathways. Gerhardt et al. provide insights into how recruited leukocytes, particularly cells of the adaptive immune system, may mediate the instability of atherosclerotic plaques in humans. Single cell RNA sequencing (scRNA-seq) and other high-parametric methodologies have advanced our understanding of cellular heterogeneity in tissues and made it possible to construct cell type atlases in an unsupervised manner. Slenders et al. describe and comment on the application of these novel tools for immunophenotyping in atherosclerosis and build associations to histo-pathological plaque features and clinical outcomes in humans. In contrast to vascular pathophysiology, Anto Michel et al. focus on the advancements in understanding cellular heterogeneity in the heart, in particular following ischemic injuries in mice and humans. Newer evidence from scRNA-seq confirms that atherosclerotic plaques are frequently populated by myeloid cells. Myeloid cell recruitment into atherosclerotic lesions is closely linked to clinical outcomes in humans. Adaptive immune cells have been shown to orchestrate and modulate myeloid cell functions and their polarization into specific macrophage subtypes with pro- and anti-inflammatory roles. von Ehr et al. provide an overview of macrophage subtypes and their roles during different stages of atherosclerotic plaque development. Platelets are crucial in the initiation of blood clots to restore vascular integrity and stop bleeding upon injury. Platelets also participate in atherothrombosis and thrombo-inflammation, two intertwined processes that ascribe these anucleate cells a central role in orchestrating acute and chronic pathological processes. Hamad et al. call attention to platelet subtypes that range from pro-coagulant and aggregatory platelets to those featuring a secretory phenotype. This report addresses how inflammatory conditions appear to differentially engage and modify distinct subpopulations of platelets.

Beyond the recruitment of specialized cell types into cardiovascular key organs, cardiometabolic risk factors and specific pro-inflammatory signaling pathways initiated by these factors represent another layer of CVD-associated inflammation addressed in our topic series. Ganesh et al. highlight how immune cells residing in the gut may impact on local and distant tissue homeostasis thus constituting a potential therapeutic target that is closely linked to metabolic alterations. Interestingly, gut-resident leukocytes are strongly affected by dietary pattern and metabolic dysregulation such as hyperglycemia or diabetes. Eventually, gut immune cells appear to relay disease-relevant signaling to promote CVD. Mammalian target of rapamycin (mTOR) signaling plays an important role in sensing and integrating the metabolic and inflammatory environment on a cellular level. The mTOR signaling complexes govern essential cellular activities including growth, proliferation, motility, energy consumption, and survival. Kaldirim et al. dissect cell-specific mTOR signaling in CVD and how it could be targeted to cease acute and chronic inflammation. Gissler et al. dissect the role of members of the Tumor Necrosis Factor associated Factors (TRAFs), which represent signaling mediators downstream of potent pro-inflammatory receptors of the TNF-superfamily. These have been postulated as potential therapeutic targets because of their potency to modulate and integrate metabolic, inflammatory, and immune signaling events.

Multiple articles of this series focus on the potential role of antigen-specific T- and B-lymphocyte responses in CVD. Recent evidence has highlighted that a multiplicity of autoreactive immune cells directed against lipids, oxidation-specific epitopes, and the myocardium may exist even in healthy individuals. Clinically, it is known that checkpoint-inhibition may re-activate some of these hibernating autoreactive T cells directed against myocardium-specific self-antigens with the capability to elicit cardiac autoimmunity and autoimmune myocarditis. Immunotherapies, particularly immune checkpoint blockade, have been a revolution in cancer treatment but their potentials in cardiovascular complications remain obscure. Nettersheim, Picard et al. highlight underlying pathways and raise attention on how immunotherapies in cancer might contribute to atheroprogression. Some of the recently described ApoB-reactive T-helper cells may originate from T_H_17 and -regulatory cells (T_reg_)—a topic summarized by Wang et al. The transcription factor Autoimmune Regulator (AIRE) is important for thymic expression of many tissue antigens and the establishment of central tolerance toward them. In an original research article, Nettersheim, Braumann et al. demonstrate that plaque development and presence of Apolipoprotein B100-reactive CD4 T cells, however, is not affected in AIRE-deficient mice. Besides T cells, that may shape the inflammatory microenvironment in plaques by secreting cytokines and direct cell-cell interactions, B cells primarily act by secreting (auto-) antibodies. The current concepts of B cell functions and subtypes in atherosclerosis are summarized by Smeets et al. Interestingly, B cell subpopulations appear to mediate both pro- and antiatherogenic functions. Furthermore, not all of these effects depend on the antibody-expressing and -secreting capacity of the B cells. The review proposes to enhance function of antigen-specific B cell clones as a potential atheroprotective therapy. Building up on this knowledge, Douna et al. demonstrate that the long-considered pro-atherosclerotic cytokine IFN-g executes anti-atherosclerotic functions by re-wiring B cells that subsequently prevent T follicular helper cell differentiation and foster T_reg_ development, thereby ameliorating atherosclerosis. Following *ex vivo* IFN-g-stimulation, B cells express the co-inhibitory molecule PD-L1 and inhibit T follicular helper cell activity. Pattarabanjird et al. uncover that the chemokine receptors CCR6 and CXCR4 are highly expressed on memory B cells associated with IgE sensitization to alpha-gal. Likewise, the authors uncover a correlation between CCR6, CXCR4, IgE expression in switched memory B cells and CAD severity. ApoB autoantibodies are thought to exert pro- and anti-atherosclerotic functions dependent on the subtype. Marchini et al. demonstrate that these autoantibodies do not associate with atherosclerotic disease, but rather with cardiometabolic risk factors, such as hypertension and obesity sparking the interesting idea that formation of auto-reactive B cells is primarily driven by metabolic alterations and does not necessarily represent a prerequisite for CVD. Our series concludes with Zhu et al., who developed a new preclinical model of aortic aneurysm formation in rats.

Overall, our topic series covers several fundamental and novel aspects of cardiovascular immunity and inflammation, an emerging field that is at the promise to build the basis for the future development of tailored personalized treatment and preventative strategies with novel immunomodulation, vaccination, and CAR T cell therapies in the future.

## Author contributions

All authors listed have made a substantial, direct, and intellectual contribution to the work and approved it for publication.

